# Fluorescence and UV–visible reflectance in the fur of several Rodentia genera

**DOI:** 10.1038/s41598-022-15952-7

**Published:** 2022-07-19

**Authors:** Gisela Sobral, Filipe Souza-Gudinho

**Affiliations:** Setor de Mamíferos, Departamento de Vertebrados, Museu Nacional/UFRJ, Quinta da Boa Vista s/n, São Cristóvão, Rio de Janeiro, RJ CEP: 20940-040 Brazil

**Keywords:** Zoology, Evolution

## Abstract

Mammals are generally brown in colour, but recent publications are showing that they may not be as uniform as once assumed. Monotremes, marsupials, and a handful of eutherians reflect various colours when lit with UV light, mostly purple. Because of these still scarce records, we aimed to explore UV reflectance among rodent genera, the most diverse mammalian group, and the group of eutherians with the most common records of biofluorescence. Here we report structures like nails and quills reflected green, but for most genera, it was faded. However, *Hystrix*, *Erethizon,* and *Ctenomys* showed intense and contrasting green glow, while *Chaetomys* presented a vivid orange anogenital. The main available explanation of fluorescence in mammals relies on porphyrin*.* This explanation applies to the cases like *Chaetomys,* where specimens showed anogenital orange biofluorescence, but does not apply to the green biofluorescence we observed. In our sample, because the structures that reflected green were all keratinized, we have reasons to believe that biofluorescence results from keratinization and is a structurally-based colouration. However, not all spines/quills equally biofluoresced, so we cannot rule out other explanations. Since Rodentia is the most common mammalian group with reports on biofluorescence, this trait likely serves various functions that match the species diversity of this group.

## Introduction

The excitation by higher energy wavelengths of light (shorter wavelength)—usually blue or UV—followed by their emission at lower energy (and longer wavelength) is called fluorescence^1^, generally resulting from green to red colours. This enigmatic mechanism is long known among vertebrates, particularly bird feathers that reflect UV light^2–4^. Subsequent works have shown that biofluorescence is widespread among animals, mainly fish^5^ and amphibians^6^. As for mammals, only marsupials were known to biofluoresce until recently^7^.

The extensive work of Pine and collaborators^7^ described that many didelphid marsupials fluoresce in all colours, and Reinhold^8^ surveyed several Australian marsupials and found that they too fluoresced. Over the past two years, several papers complemented these findings, and other mammalian groups, such as monotremes and eutherians, were included in the list of animals that biofluoresced^8–12^. Colours were most commonly pink, purple, and blue. Yellow-green fluorescence was rare and observed in only three species under a microscope, present either as a ventral stripe or lateral spots (^7^, “Metachirops mcIlhenny”; “Monodelphis adusta”; “Metachirops opossum”). The monotreme platypus *Ornithorhynchus anatinus* was the first record of pure green reflectance in mammals, with dorsal and ventral pelage appearing green under UV light^8,10^.

However, one eutherian group stands out for the frequency in biofluorescence records, the order Rodentia. Kohler et al.^10^ found that almost all examined individuals of flying squirrels *Glaucomys* spp. had pink UV reflectance, mostly pronounced ventrally, further assessed by Hughes et al.^13^. Anecdotal observations of the rodents *Melomys, Niviventer,* and *Rattus* showed that some guard hairs reflected bright blue^8,9,14^. However, biofluorescence is still not well-documented, and we know very little about how common this trait actually is. The desert-dwelling rodent springhare *Pedetes* spp. exhibit red patches along their bodies when lit with UV light^12^. While this manuscript was being written, three other papers reported several rodent species glowing at longer wavelengths spectrum when lit with a UV light. Reinhold^8^ reported that one rodent species (*Rattus rattus*) glowed green when observed with a naked eye, but the camera interpreted it as blue. Another study described gophers reflecting orange-pink and blue^14^. Moreover, Tumlison and Tumlison^15^ assessed several rodent species, and although many showed a fainted reflectance, species like Canadian beaver *Castor canadensis* presented mildly greenish guard hairs; Norway rats *Rattus norvegicus* showed green fluorescence and so did the thick underfur of muskrat *Ondatra zibethicus*.

Because rodents are the most common mammalian group known to fluoresce, we decided to widely explore fluorescence in several rodent genera, mostly from the Americas. According to the available literature, we expected to observe fluorescence in rodents inhabiting open areas, resembling *Pedetes*^12^, and tree-dwelling species, similar to *Glaucomys*^10^, and water-dwelling ones^15^. Conversely, we did not expect to observe ground-dwelling forests genera to fluoresce^5^.

## Methods

### Survey

We examined biofluorescence in several museum specimens from the order Rodentia, randomly selected, using a handheld LED UV flashlight 395 nm 100 LED. All observations were made at the mammal collection housed at Museu Nacional/UFRJ (MN/UFRJ) in Rio de Janeiro, Brazil. Animals were previously collected in several years (the earliest 1905 and the latest in 2008) and from several countries (Table 1). All specimens were dried and stuffed. For larger specimens (e.g., *Hystrix* and *Erethizon*), we washed body parts with neutral soap to clean any residue and remove any possible fungus, an organism that might reflect green^16^, and also brushed the fur so the pelage was uniform. We also provide information on the specimens regarding place and date of collection, sex, the body part in which we detected fluorescence/reflectance, and the biome in which the species occur followed^17^.

Because residual light is common when using UV light bulbs and flashlights, authors usually use a longpass yellow filter (e.g.,^10–12^). When we photographed specimens with this yellow filter, the camera did not capture the colours properly. Additionally, we did not consider purple as true biofluorescence because it was possibly a by-product of the visible purple wavelength emitted by our flashlight, or perhaps UV-reflectance. However, it is relevant to add that UV light reflection may also be an important ecological trait.

### Photographs

Upon visualising biofluorescence with a naked eye, we elected for photographs only those that reflected longer wavelengths than purple. We photographed specimens using a Nikon D7000 camera under white light and again in a dark room, where specimens were lit with UV. While taking a photograph, we moved the UV flashlight constantly to illuminate the whole specimen properly. Photo settings were apertures of f/8 (*Ctenomys*) to f/3.8 (*Hystrix*), ISOs 100, and shutter speeds between 5 s (*Hystrix* and *Erethizon*) and 30 s (*Ctenomys*).

### Photograph treatment

In order to take photographs, previous studies employed longpass filters to absorb unwanted wavelengths^5,6,10–12^. For mammals, standard excitation protocols use UV light (395 nm) and a 470 nm yellow filter attached to the camera lens, allowing green, yellow, orange, and red wavelengths to pass^10–12^.

We took photos with and without the yellow filter (LP470 Midwest Optical System Inc.), but both required processing to deal with the excess of yellow (with filter) or excess of blue (without filter). Hence, we opted not to employ the filter, but to adjust the white balance using the black-and-white scale as a reference. Additional treatment consisted of post-processing photos to remove background and debris.

## Results

**Figure 1:**
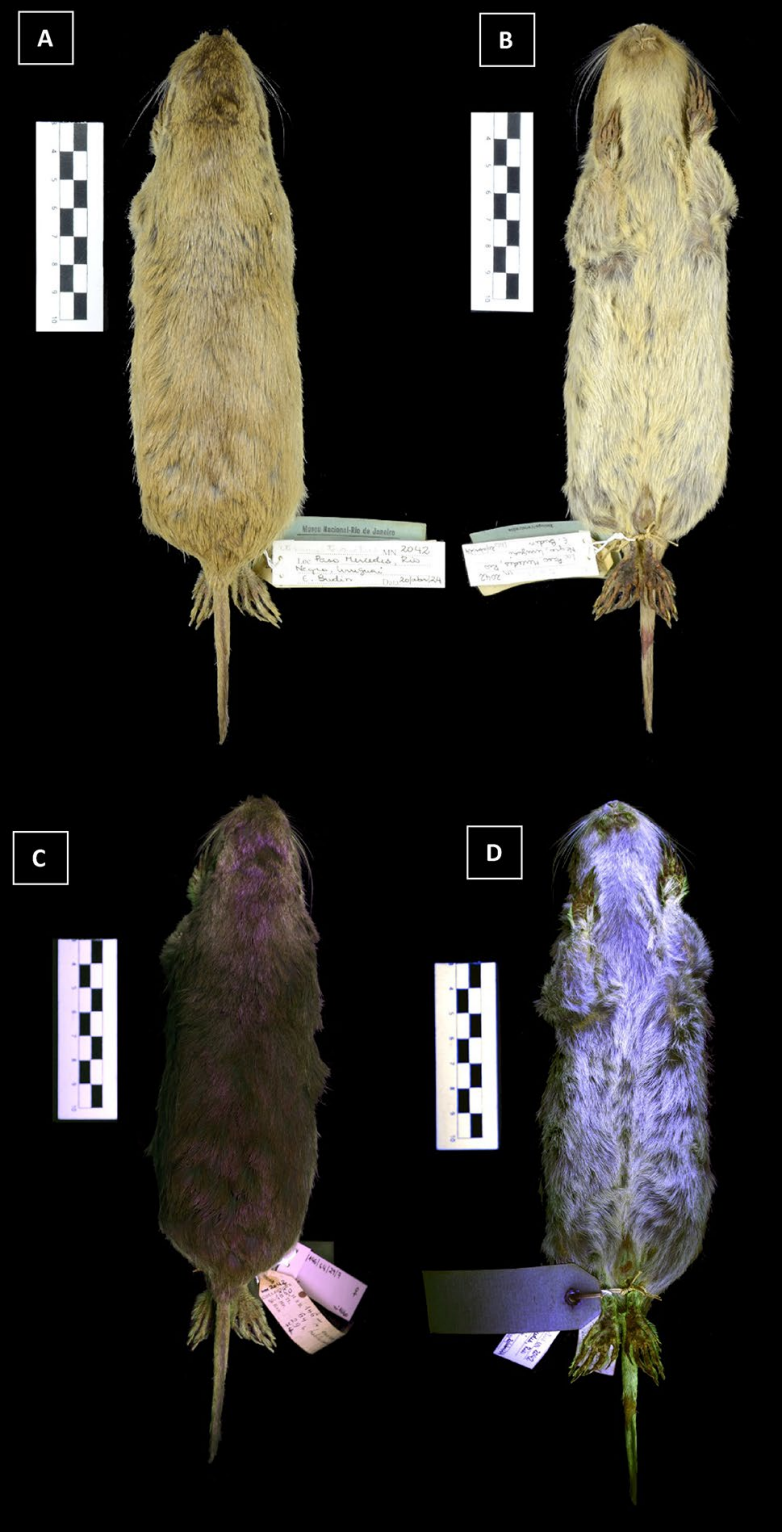
Photographs of *Ctenomys torquatus* (MN2042) under visible light (top row) and 395 nm ultraviolet (UV) light (bottom row). Dorsal (**A**, **C**) and ventral (**B**, **D**) views.

**Figure 2:**
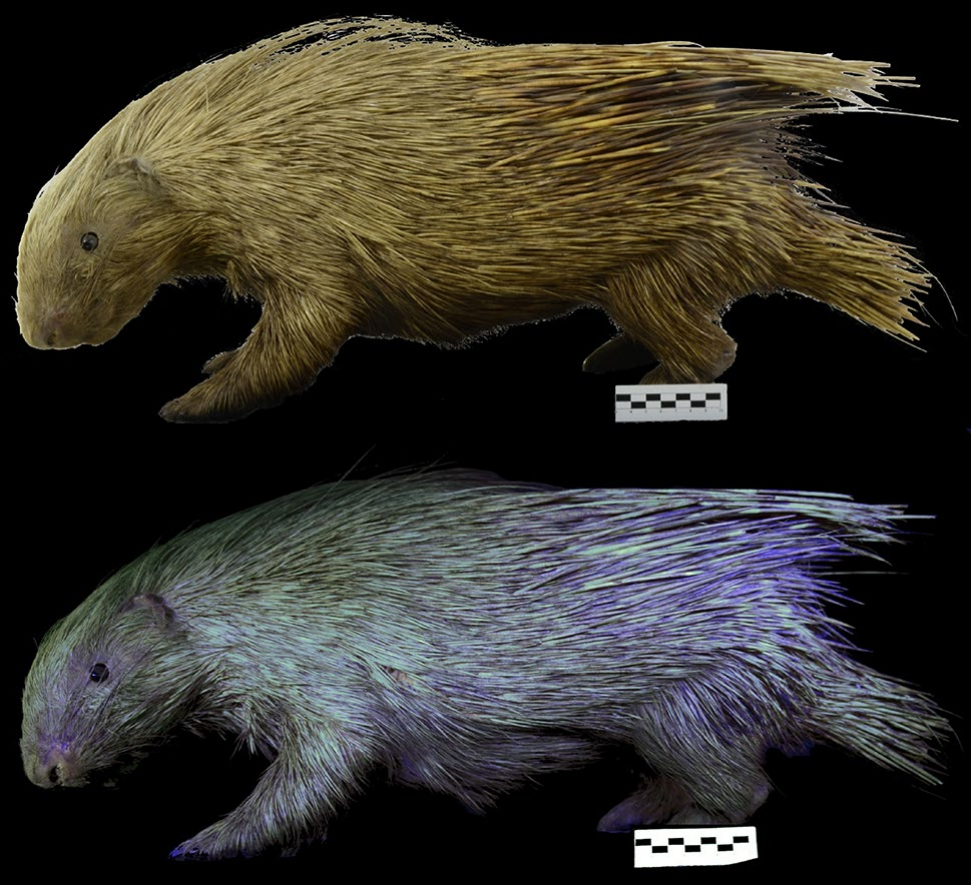
Photographs of *Hystrix javanica* (MN306) under visible light (top row) and 395 nm ultraviolet (UV) light (bottom row).

**Figure 3:**
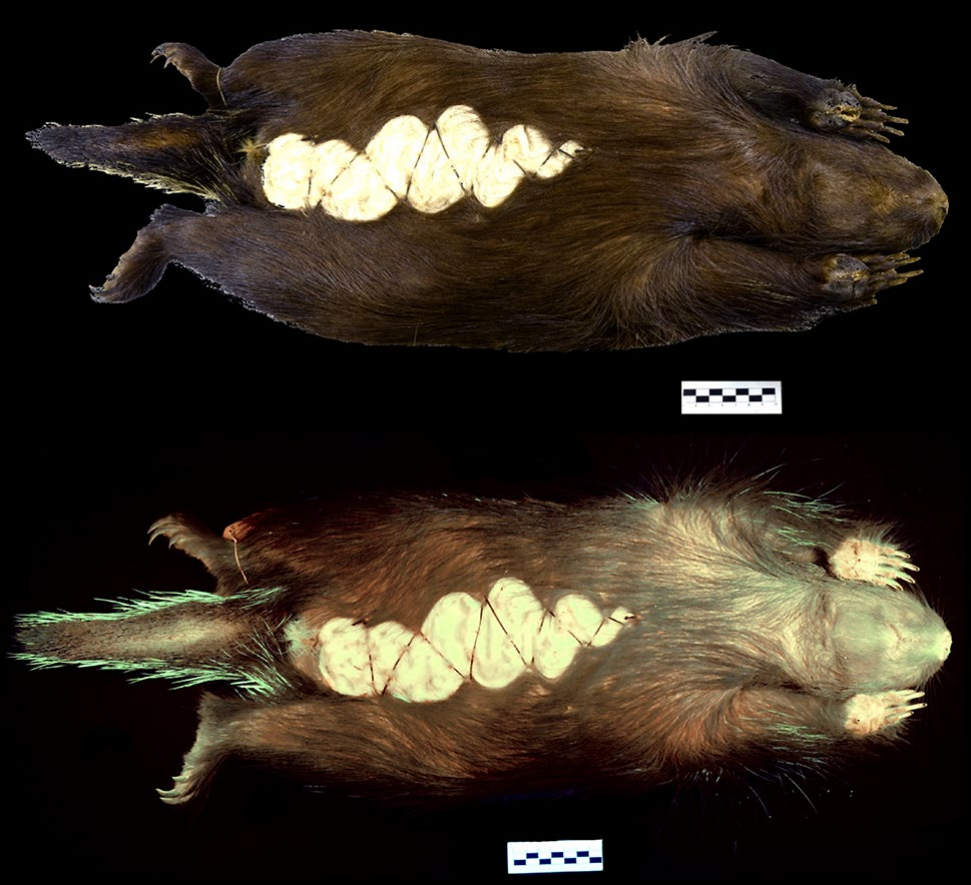
Photographs of *Erethizon dorsatum* (MN81957) under visible light (top row) and 395 nm ultraviolet (UV) light (bottom row).

We observed green (as well as other colours) UV reflectance in many of the surveyed genera (Table 1). These observations were in well-defined regions, such as the tail, perianal region, hind/forefeet fringes, and perioral hair. None of the specimens presented a patchy pattern, and colour did not depend on the angle. Nails were a common structure that glow green. While some were faded, other genera, such as *Ctenomys, Hystrix,* and *Erethizon*, glow vivid green (Figs. 1, 2, 3). All *Ctenomys* individuals were uniform in colour under white light (some pale yellow, others dark brown), but not all yellowish fur glowed green when lit with the UV flashlight. We observed green reflectance in paw hairs, vibrissae, and tail. *Hystrix* (African porcupine) showed a contrasting purple and green pattern. Since we only had one specimen, it is difficult to determine how frequent this trait is. We moved on to other porcupines expecting quills to reflect similarly. *Chaetomys, Coendou* and *Erethizon*, American porcupines, reflected quite distinctively from *Hystrix*. The back quills of the American porcupines did not reflect green, only those short ones surrounding the anogenital region and below the tail.

**Table 1:** List of genera illuminated with a handheld LED UV flashlight 395 nm, and relevant information on the surveyed specimens.

Genus	Country	Date of collection	Sex	Body part glowing	Colours	Habit	Biomes
*Akodon*	Brazil	1942–1943	5 F/5 M	Paw hair	Barely perceptible green	Te	Forest, Savannah
*Blarinomys*	Brazil	1973	4 F/6 M	–	–	SF	Forest
*Callistomys*	Brazil	1944	1 F/1 M/1 Unk	–	–	Ar	Forest
*Calomys*	Brazil	1954	5 F/5 M	–	–	Te	Forest, Savannah
*Cerradomys*	Brazil	1954	5 F/5 M	–	–	Te	Forest, Savannah
*Chaetomys*	Brazil	1939–1944	9 F/4 M/2 Unk	Anogenital/tailbase	Bright orange/green	Ar	Forest
*Clyomys*	Brazil	1986–2000	3 M/8 Unk	Belly	Faded green	SF	Open areas, savannah
*Ctenomys*	Argentina, Bolivia, Chile, Uruguay	1919, 1920, 1924, 1938, 1960	8 F/4 M/1 Unk	Paw hair	Bright green	Fs	Open areas, savannah
*Dactylomys*	Brazil	1951, 1979, 2007, 2010	5 M	–	–	Ar	Forest
*Echimys*	Brazil	1935–1966	1 F/1 M/2 Unk	–	–	Ar	Forest
*Erethizon*	USA	1905	F	Anogenital/tail/paws	Green	Ar	Forest
*Holochilus*	Brazil	1954	5 F/5 M	–	–	SA	Forest, streams
*Hystrix*	??	??	1 F	Longer quills	Green	Te	Savannah
*Isothrix*	Brazil	1919, 1934, 2000, 2002, 2004	4 F/2 M/1 Unk	–	–	Ar	Forest
*Kunsia*	Brazil	1997–2001	2 F/2 M	Nails	Green	SF	Savannah
*Lundomys*	Uruguay	1957, 1963	2 M	Nails	Green	SA	Pampas
*Makalata*	Brazil	1987, 1999, 2002, 2005	4 F/ 6 M	–	–	Ar	Forest
*Myocastor*	Brazil	1997	1 F/3 Unk	Guard hair	Faded green	SA	Forest, streams
*Neacomys*	Brazil	2007–﻿2008	3 F/11 M/2 Unk	–	–	Te	Forest
*Necromys*	Brazil	1954	5 F/5 M	–﻿	–	Te	Forest, open areas
*Nectomys*	Brazil	1954	5 F/5 M	Perioral	Green	SA	Forest, streams
*Oxymycterus*	Brazil	1954	5 F/5 M	Nails	Green	SF	Forest
*Proechimys*	Brazil	1937, 1942	4 F/6 M	Paw hair	Barely perceptible green	Te	Forest
*Rhipidomys*	Brazil	1954	5 F/5 M	–	–	Ar	Forest
*Sigmodon*	Brazil, El Salvador, Ecuador, USA	1931–1957	4 F/3 M	Perioral	Barely perceptible green	Te	Forest
*Thaptomys*	Brazil	1944–1945	3 F/6 M	–	–	Te	Forest
*Trinomys*	Brazil	1944, 1992, 2004, 2007, 2008	5 F/4 M/1 Unk	Belly	Pink blended with yellow	Te	Forest
*Wiedomys*	Brazil	1954	5 F/5 M	–	–	Sc	Savannah

Habit and biomes follow^17^. Te - terrestrial; SF - semifossorial; Ar - arboreal; Fs - fossorial; SA - semiaquatic; Sc - scansorial; Unk - unknown.

Interestingly, *Chaetomys* was quite colourful. In addition to its green tail base, its anogenital region, which was already rusty in colour under white light, presented a more evidenced orange colour under UV, contrasting with the overall dark belly. Fur in this region was softer than the rest of its body. In dorsal view, the base of its quills was bright white and contrasted with the dark quill tips.

All fur and quills that reflected green were yellow under visible light. Considering that the UV flashlight also emits a purple wavelength, we expect this combination of colours to result in brown (e.g., *Ctenomys* dorsal view), not green. Therefore, we considered green and orange reflectance as biofluorescence.

## Discussion

To our knowledge, this is only the second report of green reflectance in rodents, but the first photographically documented. Moreover, we expanded the species which fluoresce under UV light. As we expected, rodents inhabiting open areas (*Hystrix* and *Ctenomys*) fluoresced, while ground-dwelling forest species did not. Interestingly, contrary to our expectations, some species that use their claws to dig, despite inhabiting dense forests (e.g., *Kunsia*), showed fluorescent nails.

Since the substantial work of Pine et al.^7^ on mammals that can glow under UV light, reports are still scarce. Despite this scarcity, biofluorescence is present in all three major groups of mammals^7–14^. These findings indicate that reflectance occurs throughout the visible spectrum, although more frequent at lower wavelengths (blue and violet). New reports on biofluorescence (reflectance at higher wavelengths), are being published every year. However, most authors did not aim to explain the mechanisms behind the different colours.

Rodents produce porphyrin, a red-coloured photosensitive pigment^18,19^. Other chemicals have been isolated from rat fur, such as tryptophan and kynurenine, and they too are fluorescent^20,21^. Aside from pigments, keratin is a fibrous protein found in epidermic structures that glows yellow-green under UV^7,18,22,23^. Keratinised structures seem to resist the wear of digging^24^. For instance, rodents that live in dry and harsh soil environments, such as North American voles, present a keratinised epidermis layer^25^. Similarly, the naked-mole-rat, an African rodent that has an exclusively fossorial lifestyle, presents several keratinised body parts, such as their eyelids^26^. However, none of these species was observed under UV light yet. The pangolin, a non-rodent mammal, presents scales which are keratinised and reflected blue^9^, but the keratin found in pangolin scales differs from that of other mammals’ keratinised structures^27^. The paper of Millington^23^ shows that the sunlight damages the keratin fibres and is likely to make them fainter in colour. The author, however, does not mention the colour green, but yellow. More recently, Hamchand et al.^28^ proposed that bacteria present in sweat and sebaceous glands from hedgehogs, another non-rodent mammal, would be responsible for the red fluorescence they observed.

Structural colouration is produced when the light interacts with small structures (see^29^). It is mistakenly referred to as a synonym of iridescence but differs from it as colour visualisation is angle-dependent^29,30^. Because our observations did not depend on either the angle of the specimen or the observer’s, we did not consider our results as iridescence, but could potentially be structural in origin. Structural colouration is common in birds and other vertebrates (e.g.,^31^) but rare among mammals. In the few reports available, some male primates and marsupials present blue structural colour resulting from collagen arrangement^32^, while the fur scale arrangement in the back of golden moles, a non-rodent mammal, not only reflects a green sheen^33^ but is also wear-resistant^33,34^. It would be interesting to understand the microstructure of the fur and quills from the rodents presented in this paper.

Among our study species that showed green biofluorescence, *Ctenomys* is a scratch-digging/chisel-tooth rodent that could benefit from having keratinised fringes, vibrissae, and rhinarium that can resist the wear of their lifestyle. Particularly in the case of *Ctenomys*, this genus possesses comb-like hairy fringes (bristles) made of stiffened hair that edge their paws, a characteristic that gave rise to the genus name^35–37^.

*Hystrix* (African porcupine), as well as *Erethizon, Coendou,* and *Chaetomys* (American porcupines), present quills along with their bodies. However, their quills differ significantly from each other in terms of mechanical properties, structure, and function^27,38^. Some of the *Hystrix* quills are classified as true quills, which are thicker, sharper, and used for defence^39,40^. Additionally, its quills are longer, stiffer, and more resistant than the quills of American porcupines, which may explain why we did not observe the same pattern in the three genera cited above. Our findings contradict that of Hamchand et al.^28^, who did not find measurable fluorescence in *Hystrix javanica* or *Erethizon dorsatum*. We believe that keratinisation is a possible explanation for the green colour we witnessed, and we suggest that keratinisation is different for each species. However, keratinisation may not be the sole explanation for green UV reflectance, as Reinhold^8^ witnessed green fur in *Rattus* and Tumlison and Tumlison^15^ in the underfur of *Myocastor*. Additionally, the work of Hamchand et al.^28^ sheds light on the role of bacteria that biosynthesise and excrete porphyrin, a likely explanation for the red present in hedgehogs, but also the orange anogenital region of *Chaetomys* that we found.

The function of biofluorescence has been under discussion ever since its discovery. Colouration plays a vital role in communication and camouflage^10^, and UV reflection is particularly important in UV-rich environments, such as snowy^10^, and desertic areas^12^. Among golden moles, a fossorial non-rodent mammal, neither visual sexual ornamentation nor camouflage seem to account for the presence of green sheen in their fur because their fossorial habit is inconsistent with these hypotheses^34^. According to the authors, fur structure evolved to resist the wear caused by digging, and colour could be just a response to this hair scales arrangement and, perhaps, ecologically functionless. This could be the case for green reflectance we observed in our study. Because most reports on UV reflectance address rodents (^8–10,12,14^, our study), UV can be far more important to the life of rodents, serving functions that are yet to be discovered.﻿

## Data Availability

The data that support the findings of this study are available on request from the corresponding author.
